# Human sex hormone-binding globulin as a potential target of alternate plasticizers: an *in silico* study

**DOI:** 10.1186/s12900-016-0067-3

**Published:** 2016-09-30

**Authors:** Ishfaq A. Sheikh, Muhammad Yasir, Muhammad Abu-Elmagd, Tanveer A. Dar, Adel M. Abuzenadah, Ghazi A. Damanhouri, Mohammed Al-Qahtani, Mohd A. Beg

**Affiliations:** 1 King Fahd Medical Research Center, King Abdulaziz University, PO Box 80216, 21589 Jeddah, Kingdom of Saudi Arabia; 2 Centre of Excellence in Genomic Medicine Research, King Abdulaziz University, Jeddah, Kingdom of Saudi Arabia; 3 Department of Clinical Biochemistry, University of Kashmir, Srinagar, India; 4 KACST Innovation Center in Personalized Medicine, King Abdulaziz University, Jeddah, Kingdom of Saudi Arabia

**Keywords:** Docking, Sex hormone-binding globulin, DEHT, TOTM, DINCH, Endocrine disruption

## Abstract

**Background:**

Currently, alternate plasticizers are used to replace phthalate plasticizers in children’s toys, medical equipments and food packaging, due to the adverse effects of phthalate compounds on human health and laws prohibiting their use. Current information regarding the safety and potential adverse effects of alternate plasticizers is limited and recent studies have found alternate plasticizers to display similar characteristics to those observed in phthalate plasticizers. This study was undertaken to evaluate and predict the potential endocrine disrupting activity of the three most commonly used alternate plasticizers: di(2-ethylhexyl)terephthalate (DEHT), tris(2-ethylhexyl)trimellitate (TOTM), and diisononyl hexahydrophthalate (DINCH) against human sex hormone-binding globulin (SHBG) using *in silico* approaches.

**Materials and methods:**

The crystal structure of human SHBG (Id: 1D2S) was retrieved from Protein Data Bank. PubChem database was searched for the structures of alternate plasticizers, DEHT, TOTM, and DINCH. Docking was performed using Glide (Schrodinger) Induced Fit Docking module.

**Results:**

Induced Fit Docking of three alternate plasticizer compounds indicated that each of the three compounds fitted well into the steroid binding pocket of SHBG. Docking displays showed interactions of alternate plasticizers with 25–30 amino-acid residues of SHBG; 18–20 amino residues overlapped between the natural ligand, DHT, and the three compounds (commonality of 82–91 %). The hydrogen-bonding interaction of the amino-acid residue, Asn-82, of SHBG was also present in displays of DHT and all the three alternate phthalates. The binding affinity of all the three alternate phthalates was higher than DHT; maximum in DINCH followed by TOTM and DEHT.

**Conclusion:**

Our results suggested that the three alternate plasticizers have potential to engage the important interacting residues of SHBG and thus interfere in its steroid homeostatic function.

## Background

Endocrine-disrupting chemicals (EDCs) are either synthetic or natural heterogeneous compounds that are ubiquitous in the environment and perturb hormonal systems in human and animals [1–4]. Phthalate plasticizers are one such group of synthetic chemicals which have wide applications as softeners in polyvinyl chloride plastics and are used in medical and surgical equipments, consumer products, children’s toys, personal care, and common household products [5]. Exposure to phthalates in children as well as in adult men and women has been associated with many developmental and functional abnormalities of reproductive system [6–9]. High volume production and wide use of phthalates leads to widespread contamination in the environment and is therefore considered as a global public health problem.

In view of the overwhelming evidence of the adverse effects of phthalate plasticizers, the United States Congress passed a law, Consumer Product Safety Improvement Act of 2008, which has permanently banned six most toxic phthalate compounds. The Congress further mandated the convening of a Chronic Hazard Advisory Panel (CHAP) to assess further phthalate plasticizers and as of June 2015, additional phthalate compounds have been added to the list of potential prohibitions. Likewise, prohibitions on the use of phthalates have been in place in the European Union since 2005. The adverse health effects of phthalate plasticizers and prohibitions on their use has led to increased efforts in the development of alternative non phthalate compounds as plasticizers in the industry [10, 11]. There are over a dozen alternate compounds that have been used in the industry until now. Three alternate plasticizers i.e., di(2-ethylhexyl)terephthalate (DEHT), tris(2-ethylhexyl)trimellitate (TOTM), and diisononyl hexahydrophthalate (DINCH) are among the most commonly used. A higher cost in comparison to traditional plasticizers is one of the reasons that alternate phthalate plasticizers have not become popular. In spite of this, demand has increased over time with all the three aforementioned alternate plasticizers listed as high production volume chemicals for Organization of Economic Cooperation and Development countries [11]. Given the history of adverse health effects stemming from phthalate plasticizers, it is imperative that the alternate plasticizers are subjected to a great deal of scrutiny from researchers. Studies on alternate plasticizers are limited and in many cases have not found severe adverse effects. However, in some recent studies similar characteristics were found between the banned phthalate plasticizers and the aforementioned alternate plasticizers on the basis of toxicological (i.e. LD50) and developmental toxicity (no observed adverse effect levels) evaluation [12].

The alternate plasticizer DEHT is an isomer of di(2-ethylhexyl)phthalate (DEHP) possessing para carboxylic groups and is considered as a non-phthalate plasticizer [13]. Experimental studies on DEHT have produced low toxicity in rats, mice, and guinea pigs [14, 15]. Continuous dietary exposure of rats to DEHT for 2 years also indicated low levels of toxicity [16] with no evidence of reproductive and developmental abnormalities [17–19]. Toxic responses at high doses included reduced weight gain, lower food conversion efficiency, and an increase in age-related retinal degeneration rate [16]. Continuous intravenous infusion of rats with different doses of DEHT for 4 weeks did not affect their survival, body weight, and food and water consumption [20]. No hematotoxic and immunotoxic effects or histopathological lesions were found in the liver, thyroid or reproductive system. In a preliminary clinical study [13], dermal application of DEHT did not induce any adverse skin reaction. A recent study [21] in the USA suggested that DEHT was the most common plasticizer in children’s backpacks and plastic toys. Analysis on dry and wet wipes from the backpacks and toys indicated strong correlations between mass transfer of DEHT and mass content of DEHT in the products suggesting potential for human exposure through skin [21].

Another alternate plasticizer, TOTM, is an ester of trimellitic acid which is frequently used in medical equipment in Japan. The differences in structure compared to phthalate compounds lead to significant changes in the migration and extraction characteristics of this plasticizer [13, 22, 23]. TOTM has almost fully replaced DEPH in platelet storage bags because of its superior gas exchange properties [24]. Limited experimental toxicological studies have been done on TOTM. TOTM was shown as a weak estrogen in cell culture [25] and had hepatotoxic properties although lesser than DEHP [26]. Repeated dermal patch tests in human subjects resulted in lower skin sensitivity problems compared to phthalate compounds [27]. In rats, TOTM induced hepatic vascular congestion, lipid globules in hepatocytes, with significantly decreased hepatocyte mitochondrial membrane antigen immune reaction indicating liver toxicity [28]. Intravenous injection of TOTM in mice induced upregulation of 694 genes and downregulation of 974 genes in liver [29]. Further analyses of 11 genes revealed that the functions of cell cycle pathway, oxidative process and lipid metabolism were affected during hepatotoxic effects of TOTM in mice.

DINCH is a cyclohexane derivative with very low migration properties, almost 10 times lower than DEHP [13, 30], and has the advantage of similar viscosity as that of DEHP helping thus in avoiding significant changes on blending with polyvinyl chloride [31]. DINCH is a fairly recent alternate phthalate and is in use for blood tubes and nutrient solution bags. Recent data reviewed by Scientific Committee on Emerging and Newly Identified Health Risks (SCENIHR) showed that oral or dermal administration of DINCH in rats induced low toxicity, and continuous dietary exposure in rats for 28 days failed to have any effects on reproductive organs [13, 32]. Additionally, prenatal exposure and two generational tests did not produce any reproductive toxic effects. A thyroid weight increase associated with a repeated exposure for 2 years in rats was of concern and was attributed to liver problems [32]. Analysis of DINCH metabolites in the urine of adult volunteers in the United States with no known exposure to DINCH revealed that the detection rate increased from 0 % during 2000–2007 to 21 % in 2012 [33]. In another study in Germany on 300 urine samples from the German Environmental Specimen Bank, DINCH metabolites were detected in 0 % samples during 1999–2003 but the percentage of detection increased subsequently for 2006 (7), 2009 (43), and 2012 (98 %) [34]. A recent study [35] in German daycare centers revealed that DEHT and DINCH were present in 100 dust samples and 85 % of the air samples. Further, metabolites of DINCH were detectable in 100 % of the urine samples of 203 tested children from these centers.

Owing to the increasing use and demand of alternate plasticizers, there is an urgent need for exhaustive studies on their potential adverse effects on human health. It is imperative that all possible methods including epidemiological, clinical, in vivo and in vitro experimental approaches, and *in silico* predictive studies are utilized to maximize the information on the potential health effects of alternate plasticizers. Disruption of hormone signaling pathways is commonly thought to be the mechanism through which many of the environmental contaminants exert their effects on human and animal systems [1]. Sex hormone-binding globulin (SHBG) is a high molecular weight steroid binding circulatory protein involved in maintaining the androgen and estrogen homeostasis in the body [36]. The SHBG has also been reported to bind with phthalates and other environmental contaminants [37–39] and, thus, is a potential target for alternate plasticizer compounds in the body. Computational studies of alternate plasticizers with peroxisome proliferating-activated receptors (PPARs) [40, 41] showed no or weak interactions. Computational studies of alternate plasticizers with nuclear receptors or steroid binding carrier proteins are not available. SHBG was chosen for *in silico* studies with alternate plasticizers in this initial study based on our recent computational study [42] that showed high binding affinity of SHBG with phthalate plasticizers.

This study was undertaken to investigate the structural binding characteristics of the three alternate plasticizers, DEHT, TOTM, DINCH with SHBG using *in silico* approaches. It was expected that the computational systems approach-derived binding mechanisms, the distinctive binding pattern, and interacting residues of SHBG will help in predicting potential endocrine disrupting risks of the three alternate plasticizers which are already on the market.

## Methods

**Fig. 1:**
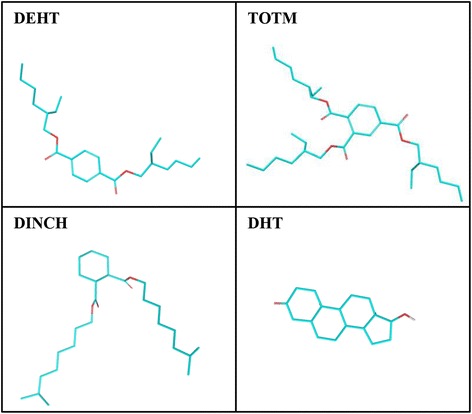
Two dimensional representation of three alternate phthalate ligands, di(2-ethylhexyl)terephthalate (DEHT), tris(2-ethylhexyl)trimellitate (TOTM), and diisononyl hexahydrophthalate (DINCH), and natural ligand dihydrotestosterone (DHT)

Schrodinger 2015 suite with Maestro 10.3 (graphical user interface) software (Schrodinger, LLC, New York, NY, 2015) was used for docking studies of DEHT, TOTM, and DINCH with SHBG. The two dimensional structures of the three alternate plasticizers are shown (Fig. 1) and their abbreviations and PubChem compound identities (CIDs) are presented (Table 1).

**Table 1:** Nomenclature, commonly used abbreviations, and PubChem IDs of the three alternate plasticizers for docking study with human sex hormone-binding globulin (SHBG)

S.No.	Name	Abbreviation	PubChem ID
1	Di(2-ethylhexyl)terephthalate	DEHT	22932
2	Tris(2-ethylhexyl)trimellitate	TOTM	18752
3	Diisononyl hexahydrophthalate	DINCH	11524680
4	Dihydrotestosterone	DHT	10635

### Protein selection and preparation

The Protein Data Bank (PDB; http://www.rcsb.org/) was used to retrieve the crystal structure of human SHBG (PDB code: 1D2S) at 1.55 Å. resolution. The SHBG was co-complexed with its natural ligand, dihydrotestosterone (DHT). The co-complex crystal structure was prepared by protein preparation wizard workflow of Schrodinger after importing it into the docking software, Glide (Schrodinger suite 2015–3; Schrodinger, LLC). Removal of water molecules and addition of hydrogen atoms in the crystal structure was followed by making loops and missing side chains by Prime 3.0 module. Optimization of H-bond particularly for Asp, Glu, and His hydroxyl containing residues was done. Optimization of the hydrogen bonding network was performed and OPLS_2005 force field was used for a geometry optimization to a maximum root-mean-square deviation (RMSD) of 0.30 Å. The bound ligand, DHT in crystal complex was selected and used for docking of DEHT, TOTM, and DINCH and grid boxes were generated.

### Ligand preparation and conformational search

Maestro 10.3 (Maestro, version 10.3, Schrodinger, LLC) was used to draw ligand structures (Fig. 1). Ligands were prepared using LigPrep module (Schrodinger 2015: LigPrep, version 3.1, Schrodinger, LLC). Correct molecular geometries and ionize at biological pH 7.4 were obtained by using the OPLS-2005 force field software.

### Induced fit docking

Schrodinger’s Induced Fit Docking (IFD) module was used for docking analyses of the three alternate plasticizer compounds i.e. DEHT, TOTM, and DINCH. LigPrep module was used to prepare the ligands and were submitted as starting geometries to IFD. The IFD has the ability of sampling the minor changes in the backbone structure as well as robust conformational changes in side chains [43]. A softened-potential docking is performed in the first IFD stage where docking of the ligand occurs into an ensemble of the binding protein conformations. Subsequently, complex minimization for highest ranked pose is performed where both the ligand and binding sites are free to move.

### Binding energy calculations

The ligand binding affinity calculations against the crystal complex was done using Prime module of Schrodinger 2015 with MMGB-SA function.

## Results

**Fig. 2:**
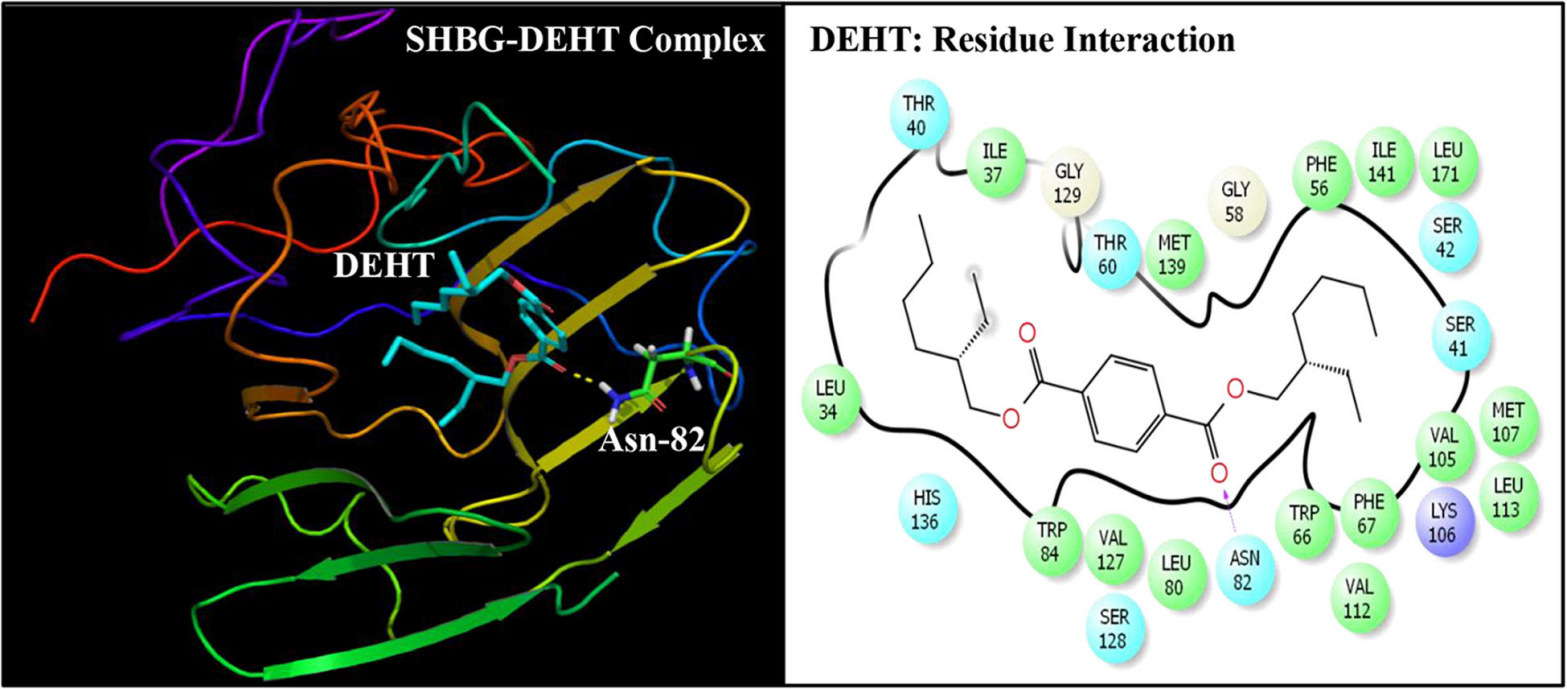
Ribbon form representation of docking complex of sex hormone-binding globulin (SHBG) with di(2-ethylhexyl)terephthalate (DEHT) (*left panel)*. Amino-acid residues in the binding pocket of SHBG involved in interactions with DEHT (*right panel*)

**Fig. 3:**
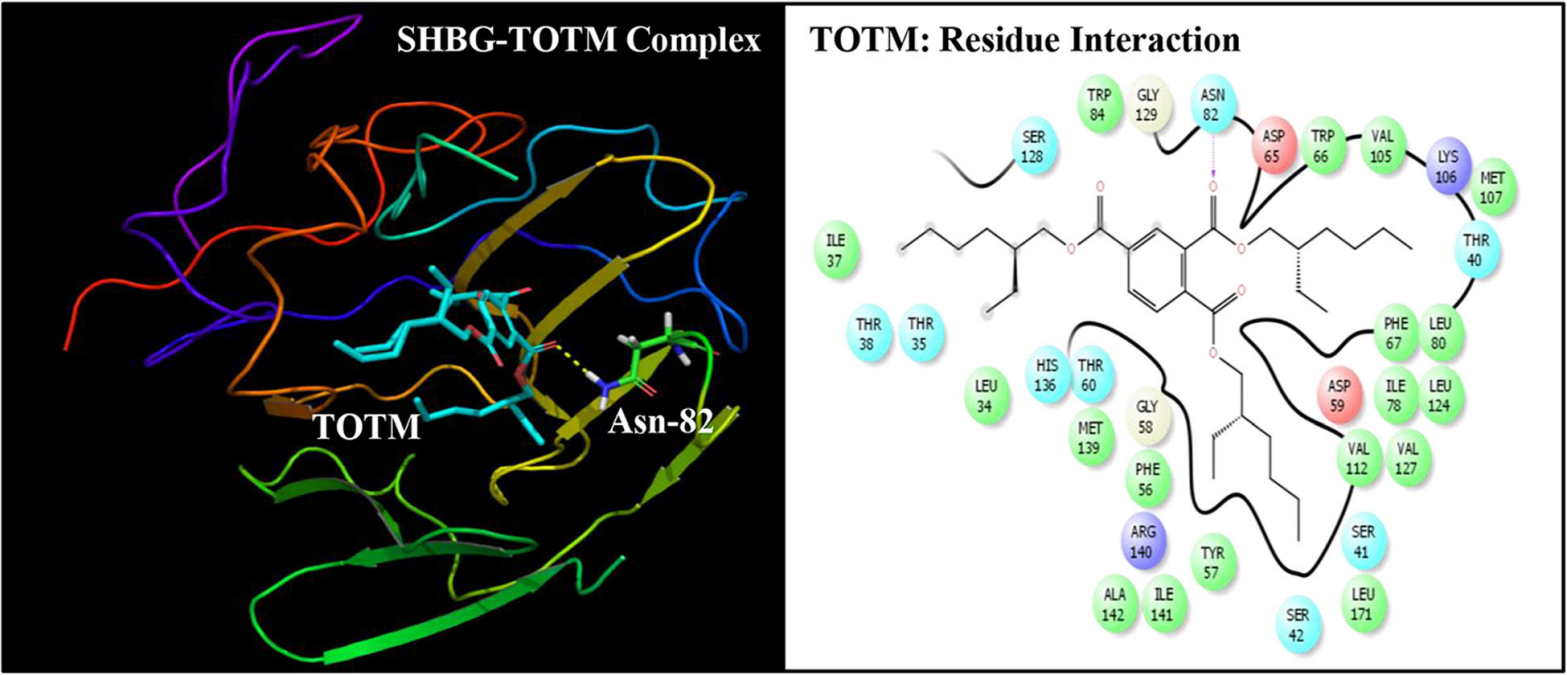
Ribbon form representation of docking complex of sex hormone-binding globulin (SHBG) with tris(2-ethylhexyl)trimellitate (TOTM) (*left panel*). Amino-acid residues in the binding pocket of SHBG involved in interactions with TOTM (*right panel*)

**Fig. 4:**
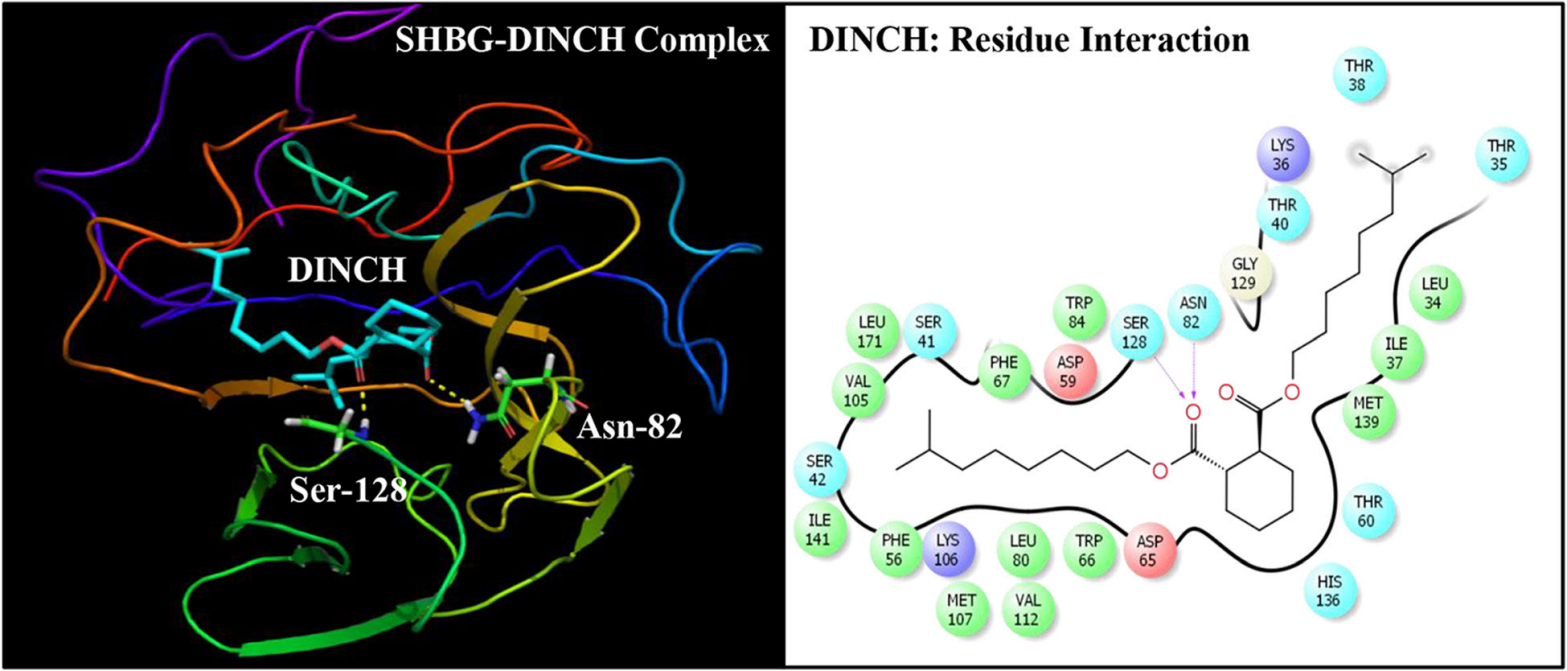
Ribbon form representation of docking complex of sex hormone-binding globulin (SHBG) with diisononyl hexahydrophthalate (DINCH) (*left panel*). Amino-acid residues in the binding pocket of SHBG involved in interactions with DINCH (*right panel*)

**Fig. 5:**
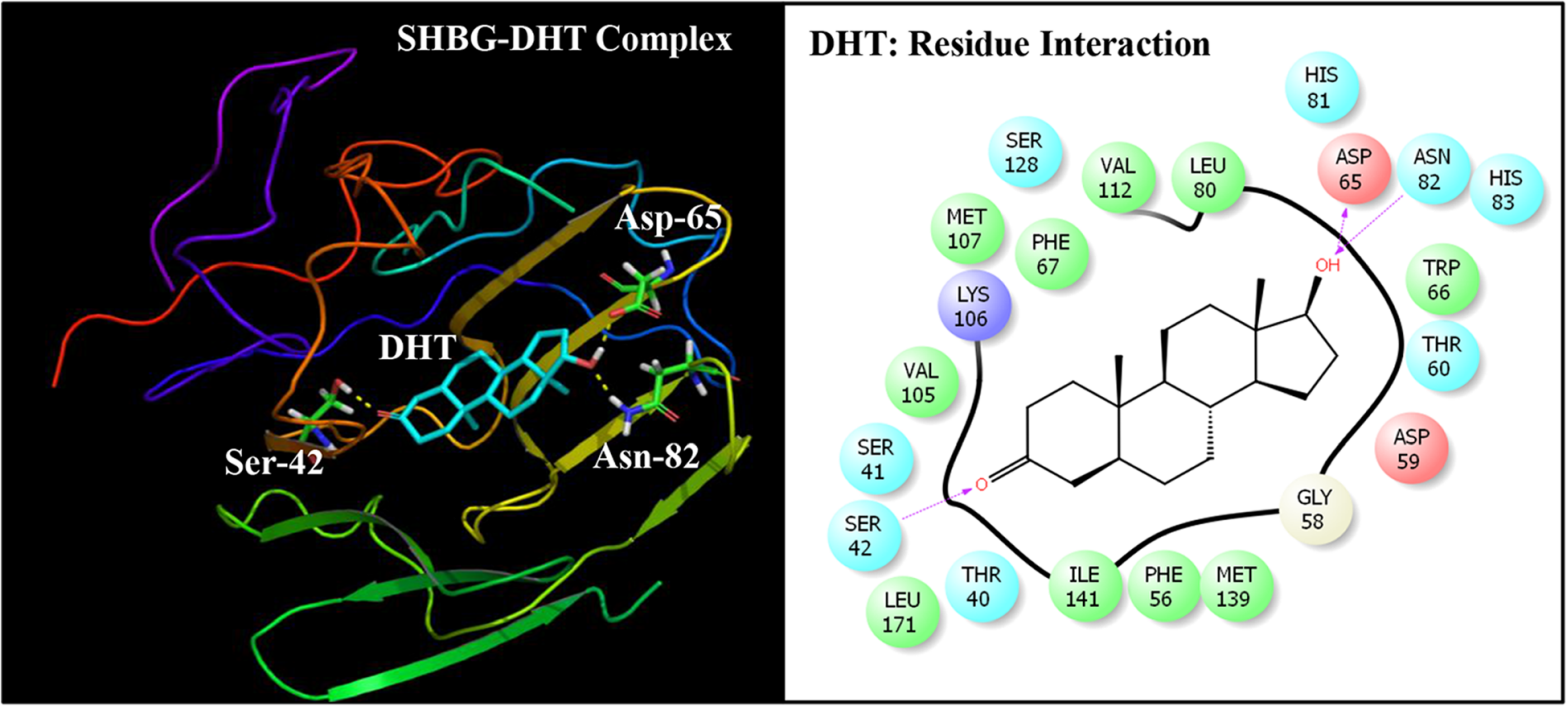
Overall all ribbon form representation of sex hormone-binding globulin (SHBG) co-complex with natural ligand, dihydrotestosterone (DHT) (*left panel*). Amino-acid residues in the binding pocket of SHBG involved in interactions with DHT (*right panel*)

Multiple docking poses were generated when the three alternate phthalate compounds i.e. DEHT, TOTM, and DINCH were subjected to docking simulation by successful execution of IFD against the hormone binding pocket of SHBG. The best pose for each of three compounds was utilized for further computational analyses (Figs. 2, 3 and 4). Similarly, the best pose of the bound native ligand, DHT was used for analyses after the IFD (Fig. 5).

### Molecular docking of DEHT with SHBG

The DEHT docking complex with SHBG and corresponding interacting amino-acid residues are shown (Fig. 2). The docking complex shows that DEHT interacted with 25 SHBG residues (Leu-34, Ile-37, Thr-40, Ser-41, Ser-42, Phe-56, Gly-58, Thr-60, Trp-66, Phe-67, Leu-80, Asn-82, Trp-84, Val-105, Lys-106, Met-107, Val-112, Leu-113, Val-127, Ser-128, Gly-129, His-136, Met-139, Ile-141, Leu-171) in the binding pocket of SHBG. The DEHT formed a hydrogen bond against residue, Asn-82 of SHBG (Fig. 2). The Dock score, Glide score, and the binding affinity were similar to those of the bound native ligand, DHT, with SHBG (Table 2).

**Table 2:** Number of interacting residues, number and percentage of residues common with dihydrotestosterone (DHT), Dock score, Glide score and binding affinity values (MMGB-SA values) of di(2-ethylhexyl)terephthalate (DEHT), tris(2-ethylhexyl)trimellitate (TOTM), diisononyl hexahydrophthalate (DINCH), and natural ligand, DHT, after Induced Fit Docking with human sex hormone-binding globulin (SHBG)

S. No.	Ligand	Number of interacting residues	Number of interacting residues common with DHT (%)	Docking score (Kcal/mol)	Glide score (Kcal/mol)	MMGB-SA (Kcal/mol)
1	DEHT	25	18 (82 %)	−8.65	−8.65	−130.83
2	TOTM	30	18 (82 %)	−9.24	−9.24	−146.21
3	DINCH	30	20 (91 %)	−10.16	−10.16	−166.83
4	DHT	22	22 (100 %)	−12.02	−12.02	−129.89

The complex of natural ligand, DHT, and SHBG and the corresponding interacting amino-acid residues are shown (Fig. 5). The complex shows that 22 amino-acid residues of SHBG (Thr-40, Ser-41, Ser-42, Phe-56, Gly-58, Asp-59, Thr-60, Asp-65, Trp-66,Phe-67, Leu-80, His-81, Asn-82, His-83, Val-105, Lys-106, Met-107, Val-112, Ser-128, Met-139, ILe-141, Leu-171) interacted with DHT. Residues Ser-42, Asp-65, and Asn-82 formed 3 hydrogen bonds with DHT. The comparison of the docking complex of DEHT-SHBG with interacting residues of natural SHBG ligand, DHT, showed that 18 DEHT interacting residues overlapped.

### Molecular docking of TOTM with SHBG

The TOTM docking complex with SHBG and corresponding interacting amino-acid residues are shown (Fig. 3). The docking complex shows that TOTM interacted with 30 SHBG residues (Leu-34, Thr-40, Ser-41, Ser-42, Phe-56, Gly-58, Asp-59, Thr-60, Phe-67, Ile-78, Leu-80, Asn-82, Trp-84, Val-105, Lys-106, Met-107, Gly-109, Asp-110, Val-112, Ser-111, Leu-113, Val-127, Ser-128, Leu-124, Gly-129, His-136, Met-139, Ile-141, Trp-170, Leu-171) in the binding pocket of SHBG. The TOTM formed a hydrogen bond against residue, Asn-82 of SHBG (Fig. 3). The Dock score and Glide score were lower, however, the binding affinity was higher than that of the natural ligand, DHT, with SHBG (Table 2). The comparison of the docking complex of TOTM-SHBG with interacting residues of natural SHBG ligand, DHT, showed that 18 DEHT interacting residues overlapped (Fig. 5).

### Molecular docking of DINCH with SHBG

The DINCH docking complex with SHBG and corresponding interacting amino-acid residues are shown (Fig. 4). The docking complex shows that DINCH interacted with 30 SHBG residues (Leu-34, Thr-35, Lys-36, Ile-37, Thr-38, Thr-40, Ser-41, Ser-42, Phe-56, Gly-58, Asp-59, Thr-60, Asp-65,Trp-66, Phe-67, Leu-80, Asn-82, Trp-84, Val-105, Lys-106, Met-107, Val-112, Leu-113, Val-127, Ser-128, Gly-129, His-136, Met-139, ILe-141, Leu-171) in the binding pocket of SHBG. The DINCH formed two hydrogen bonds with residues, Asn-82 and Ser-128 of SHBG (Fig. 4). The Dock score and Glide score were lower, however, the binding affinity was higher than that of the natural ligand, DHT, with SHBG (Table 2). The comparison of the docking complex of TOTM-SHBG with interacting residues of natural SHBG ligand, DHT, showed that 20 DEHT interacting residues overlapped (Fig. 5).

### Comparison among Ligands

The IFD complexes of the three alternate phthalates i.e., DEHT, TOTM, and DINCH with SHBG showed interactions with 25–30 amino-acid residues (indicated above for each ligand). There were 18–20 amino residues overlapping between the natural ligand, DHT, and the three alternate phthalate compounds (commonality of 82–91 %; Table 2). Seventeen amino-acid residues of SHBG (Thr-40, Ser-41, Ser-42, Phe-56, Gly-58, Thr-60, Phe-67, Leu-80, Asn-82, Val-105, Lys-106, Met-107, Val-112, Ser-128, Met-139, ILe-141, Leu-171) were overlapping among the DHT and all the three alternate phthalates (Table 3). Additionally, two amino residues were overlapping among DHT and 2 of the 3 alternate phthalates (Table 3). The hydrogen bonding interaction of the amino-acid residue, Asn-82, of SHBG was also present in displays of DHT and all the three alternate phthalates. Five SHBG interacting residues (Trp-84, Leu-113, Val-127, Gly-129, and His-136) were common among the three alternate phthalates but not for DHT. The Dock score and Glide score were highest in DHT and progressively decreased for DINCH, TOTM, and DEHT. However, the binding affinity of all the three alternate phthalates was higher than DHT; maximum in DINCH followed by TOTM and DEHT (Table 2).

**Table 3:** Amino-acid residues of human sex hormone-binding globulin (SHBG) that were common among co-complex natural ligand, dihydrotestosterone (DHT), and alternate plasticizers, di(2-ethylhexyl)terephthalate (DEHT), tris(2-ethylhexyl)trimellitate (TOTM), and diisononyl hexahydrophthalate (DINCH)

S.No.	Common interacting residues	S.No.	Common interacting residues
1	Thr-40	11	Asn-82
2	Ser-41	12	Val-105
3	Ser-42	13	Lys-106
4	Phe-56	14	Met-107
5	Gly-58	15	Val-112
6	Asp-59^a^	16	Ser-128
7	Thr-60	17	Met-139
8	Trp-66^b^	18	ILe-141
9	Phe-67	19	Leu-171
10	Leu-80		

Amino-acid residue indicated by superscript (^a^) was not shared by DEHT and the residue indicated by superscript (^b^) was not shared by TOTM

## Discussion

Human SHBG is an androgen and estrogen binding circulatory protein synthesized in liver [36, 44]. The majority of plasma dihydrotestosterone, testosterone, and estradiol are bound to SHBG which maintains their bioavailability equilibrium and protects them from metabolic degradation [36]. Only a minor (1–3 %) portion of the steroid hormones are in free form in the plasma and this free form is considered bioavailable or “active” form for the target receptors.

The IFD of three alternate plasticizer compounds with human SHBG in this study indicated that each of the three compounds fitted well into the steroid binding pocket of SHBG. The high dock scores and high binding affinity values indicated that the docking complexes of the alternate plasticizer compounds and SHBG were in their most favorable conformation. The docking analyses revealed that several important SHBG residues exerted hydrophobic and hydrogen-bonding interactions with each of the alternate plasticizer compounds and contributed to the stability of the docking complex. About 82–91 % of the SHBG interacting residues for the bound ligand, DHT, overlapped with SHBG interacting residues for the three alternate plasticizers, which suggested structural similarity to a common steroid scaffold and explained the binding of the compounds to the steroid binding pocket of SHBG. Seventeen interacting residues of SHBG and hydrogen bonding interaction with residue Asn-82 were common among the DHT and each of the three alternate phthalates. The high commonality of the SHBG interacting residues between native ligand, DHT, and three alternate plasticizers together with high dock score and high binding affinity suggested high potential for interference with the native steroid to SHBG binding function. The three alternate plasticizers i.e. DEHT, TOTM, and DINCH showed high binding affinity with SHBG that was higher than that for the native bound ligand, DHT, indicating more tight interactions with the SHBG. This suggested that the indicated alternate plasticizers bound to the steroid binding site of the SHBG more tightly than the native ligand, DHT, and thus each of the alternate plasticizers has potential to inhibit the steroid binding function of SHBG strongly by engaging the important residues. Therefore, these alternate plasticizers, on a preliminary basis, could possibly disrupt the androgen and estrogen homeostasis of SHBG more potently and thus, interfere with steroid signaling function.

This study is, to our knowledge, the first structure based study of alternate plasticizers showing interaction with human SHBG. Comparative molecular field analysis (CoMFA) and molecular similarity indices in a comparative analysis (CoMSiA) have been used to show the binding simulations for a large number of steroid and non-steroid compounds with human and zebrafish homolog of SHBG [39, 45–47]. But, none of previous docking simulation studies apparently included the three indicated alternate plasticizers DEHT, TOTM, and DINCH. In addition, to our knowledge, in vitro competitive binding of alternate plasticizers with SHBG has also not been performed. However, docking studies involving the three indicated alternate plasticizers with PPARs have been reported [40, 41]. In the first study [40], TOTM was not found to fit into the receptor binding pocket of PPAR-α and -γ and, therefore, failed to interact with PPAR receptors suggesting TOTM to be a safer alternative to phthalate plasticizers. In the subsequent study [41], DEHT, TOTM, and DINCH were reported to have varied *in silico* binding ranging from no binding to very weak binding with PPARs, again suggesting a low potential risk of endocrine disruption. However, PPARs are only one of the several possible ways through which the EDCs can exert their action in the human body [1]. Other enzymatic, nonnuclear receptor, and binding protein pathways (including that of SHBG) regulating the development and function of male and female reproductive system have also been proposed to mediate the endocrine disrupting activity [1, 48]. Additionally, even though the parent compound may not apparently show any prediction for potential side effects, yet, it may be the metabolites of the compounds that are potent endocrine disruptors. For example, in a recent study [49], rat primary stromal vascular fraction (SVF) of adipose tissue did not show any differentiation on treatment with DINCH. However, treatment with MINCH (monoester metabolite of the DINCH) induced the differentiation of SVF preadipocytes into mature adipocytes indicating MINCH to be a potent disruptor of PPAR-α. Further, the interactions of even the weakly bound EDCs with SHBG may be of high physiological importance especially during the prepubescent period when estradiol and testosterone levels are low [50, 51] and during pregnancy [52] or when taking medications (use of contraceptives [53]) when SHBG levels are high. These EDCs even in low levels can bind to greater available binding sites of SHBG and interfere in hormone homeostasis and bioavailability of estrogens and androgens. In this regard, it is of high importance to perform in vitro binding studies as well as endogenous steroid displacement studies for the three indicated alternate plasticizers with human SHBG to confirm and support the *in silico* findings of high binding affinity in the present study. In addition, future in vivo studies in laboratory animals such as mice on the clearance rate and ovarian and testicular accumulation of the labelled compounds in response to human SHBG are also suggested.

The current study is an attempt to understand the molecular interactions of alternate plasticizers with human SHBG and to help in predicting the risk of potential disruption in the steroid homeostasis of the human body. Limited experimental studies on the toxicology of the three indicated alternate plasticizers are available [10, 11, 13, 54] and epidemiological and clinical studies on the indicated alternate plasticizers are almost negligible. However, due to the prohibitions and increasing restrictions on the use of phthalate plasticizers, there is an increasing utilization of the indicated alternate plasticizers for the industrial and personal use. This is evidenced by the increased volume of the production of these compounds and increased applications in children’s products in recent years [11]. The increased environmental contamination and exposure risks with alternate plasticizers were indicated by studies that have shown an increasing detection rate of plasticizers such as DINCH in adult volunteers from 0 % in 2007 to 21 % in 2012 [33] in the USA. Similarly, detection rate of DINCH metabolites increased from 0 % samples during 1999–2003 to 98 % samples in 2012 in 300 urine samples from the German Environmental Specimen Bank [34]. Although, the available limited studies indicate a low toxicological profile of the indicated three alternate plasticizers, there is a lack of definitive information regarding the metabolic pathways, carcinogenic profile and endocrine disruption risk. In order to help allay increasing fears of the risks surrounding alternate plasticizers, we would recommend more experimental studies to unravel the pathways of exposure, more data on human exposure with information on the adverse effects on highly susceptible demographic groups such as pregnant women, infants and children, and an overall safety assessment comparing alternative plasticizers to traditional phthalate plasticizers. As the use of alternate plasticizers becomes more widespread, the potential risks will increase suggesting that further studies must be conducted as a matter of urgency.

## Conclusions

The present structural binding study on DEHT, TOTM, and DINCH was expected to help in predicting potential endocrine disrupting risks of the three alternate plasticizers against SHBG. Induced Fit Docking of three alternate plasticizer compounds indicated that each of the three compounds fitted well into the steroid binding pocket of SHBG. About 82–91 % of the SHBG interacting residues for the bound ligand, DHT, overlapped with SHBG interacting residues for the three alternate plasticizers. The hydrogen-bonding interaction of the amino-acid residue, Asn-82, of SHBG was also present in displays of DHT and all the three alternate phthalates. The Dock score and Glide score were highest in DHT and progressively decreased for DINCH, TOTM, and DEHT. However, the three alternate plasticizers showed high binding affinity with SHBG that was higher than that for the native bound ligand, DHT, indicating more tight interactions with the SHBG. Thus, on a preliminary basis, there is a high risk of these alternate plasticizers i.e. DEHT, TOTM, and DINCH binding to the SHBG in the circulation and potentially displacing the endogenous testosterone and estradiol leading to potential disruption of the androgen-estrogen homoeostasis in the body.

## Abbreviations

CHAP: Chronic Hazard Advisory Panel
CIDs: PubChem compound identities
CoMFA: Comparative molecular field analysis
CoMSIA: Comparative molecular similarity index analysis
DEHP: di-(2-ethylhexyl)phthalate
DEHT: di(2-ethylhexyl)terephthalate
DHT: Dihydrotestosterone
DINCH: di-isononyl hexahydrophthalate
EDCs: Endocrine-disrupting chemicals
IFD: Induced Fit Docking
MINCH: Monoester metabolite of DINCH
PDB: Protein Data Bank
PPARs: Peroxisome proliferating-activated receptors
RMSD: Root-mean-square deviation
SCENIHR: Scientific Committee on Emerging and Newly Identified Health Risks
SHBG: Sex hormone binding globulin
SVF: Stromal vascular fraction
TOTM: Tris(2-ethylhexyl)trimellitate
